# A Bivalent Recombinant* Mycobacterium bovis* BCG Expressing the S1 Subunit of the Pertussis Toxin Induces a Polyfunctional CD4^+^ T Cell Immune Response

**DOI:** 10.1155/2019/9630793

**Published:** 2019-02-28

**Authors:** Alex I. Kanno, Cibelly Goulart, Luciana C. C. Leite, Ana C. Pagliarone, Ivan P. Nascimento

**Affiliations:** Laboratório de Biotecnologia Molecular IV, Laboratório Especial de Desenvolvimento de Vacinas, Instituto Butantan, São Paulo, SP, Brazil

## Abstract

**Background:**

A recombinant BCG strain expressing the genetically detoxified S1 subunit of pertussis toxin 9K/129G (rBCG-S1PT), previously constructed by our research group, demonstrated the ability to develop high protection in mouse models of pertussis challenge which correlated with the induction of a Th1 immune response pattern. The Th1 immune response induced by rBCG-S1PT treatment was also confirmed in the murine orthotopic bladder cancer model, in which the intravesical instillation of rBCG-S1PT resulted in an improved antitumor effect. Based on these observations, we hypothesize that the reengineering of the S1PT expression in BCG could increase the efficiency of the protective Th1 immune response in order to develop a new alternative of immunotherapy in bladder cancer treatment.

**Objectives:**

To construct rBCG strains expressing S1PT from extrachromosomal (rBCG-S1PT) and integrative vectors (rBCG-Sli), or their combination, generating the bivalent strain (rBCG-S1+S1i), and to evaluate the respective immunogenicity of rBCG strains in mice.

**Methods:**

Mycobacterial plasmids were constructed by cloning the* s1pt* gene under integrative and extrachromosomal vectors and used to transform BCG, individually or in combination. Antigen expression and localization were confirmed by Western blot. Mice were immunized with wild-type BCG or the rBCG strains, and cytokines quantification and flow cytometry analysis were performed in splenocytes culture stimulated with mycobacterial-specific proteins.

**Findings:**

S1PT expression was confirmed in all rBCG strains. The extrachromosomal vector directs S1PT to the cell wall-associated fraction, while the integrative vector directs its expression mainly to the intracellular fraction. Higher levels of IFN-*γ* were observed in the splenocytes culture from the group immunized with rBCG-S1i in comparison to BCG or rBCG-S1PT. rBCG-S1+S1i showed higher levels of CD4^+^ IFN-*γ*^+^ and double-positive CD4^+^ IFN-*γ*^+^ TNF-*α*^+^ T cells.

**Conclusions:**

rBCG-S1+S1i was able to express the two forms of S1PT and elicited higher induction of polyfunctional CD4^+^ T cells, indicating enhanced immunogenicity and suggesting its use as immunotherapy for bladder cancer.

## 1. Introduction

The Bacillus Calmette-Guérin (BCG) vaccine was developed almost a century ago and since then has been used against tuberculosis being the responsible for saving millions of lives worldwide. In addition to tuberculosis control, the benefits of BCG vaccination are also related to nonspecific protection against other infectious diseases [1]. BCG is also a well-known agent for the therapeutic treatment of bladder cancer. The intravesical instillation with BCG has been widely used as clinical treatment against superficial bladder carcinoma* in situ* for decades, reaching 60% of effectiveness [2]. Although the antitumor mechanisms of BCG are complex, it is well established that a Th1 profile with production of proinflammatory cytokines such as IFN-*γ* and TNF-*α* is correlated with the protective action and the success of the treatment [2, 3].

Several studies used BCG as a live vector to express a variety of viral, bacterial, and parasite antigens [4]. rBCG strains has been generated by the expression of antigens through a variety of different strategies [5] including dual promoters [6], fused antigens [7], multiple integrations into the mycobacterial genome [8], and promoter engineering [9] or as an operon [10]. It was demonstrated that rBCG strains expressing Th1 cytokines induced higher cytotoxicity of PBMCs* in vitro* against bladder tumor cell lines [11, 12]. In the murine orthotopic bladder cancer model, mice treated with rBCG secreting IFN-*γ* showed higher survival rates in comparison to mice treated with BCG carrying the empty vector [13].

Previous work in our laboratory led to the construction of a recombinant BCG strain expressing the genetically detoxified S1 subunit of pertussis toxin 9K/129G (rBCG-S1PT) for use as a neonatal vaccine against pertussis. This vaccine showed promising results in the protection against an intracerebral challenge with lethal dose of* Bordetella pertussis*. Furthermore, mice immunized with rBCG-S1PT also showed induction of an increased Th1 immune response against mycobacterial proteins in comparison to mice immunized with wild-type BCG [14–16]. Based on these studies, our group evaluated the therapeutic application of rBCG-S1PT in an orthotopic model of murine bladder cancer. In comparison to wild-type BCG, rBCG-S1PT induced increased expression of TNF-*α* and IL-10, promoted the reduction of bladder tumor development, and showed higher survival of animals [17, 18].

Since the increased antitumor activity of rBCG-S1PT was related to its ability to induce an effective Th1 immune response, we hypothesize that the differential expression of S1PT could improve the immunotherapeutic effectiveness of rBCG. The aim of this work was to construct and evaluate the immunogenicity of rBCG strains expressing S1PT through single (extrachromosomal or integrative vectors) and bivalent expression systems (combination of both single expressions).

## 2. Material and Methods

### 2.1. Ethics

Female BALB/c mice (5 to 8 weeks old) were supplied by the Animal Housing Facility of the Butantan Institute and housed under adequate conditions according to the ethical committee. This study was approved under the protocol 1178/14.

### 2.2. Cloning Procedure

**Figure 1:**
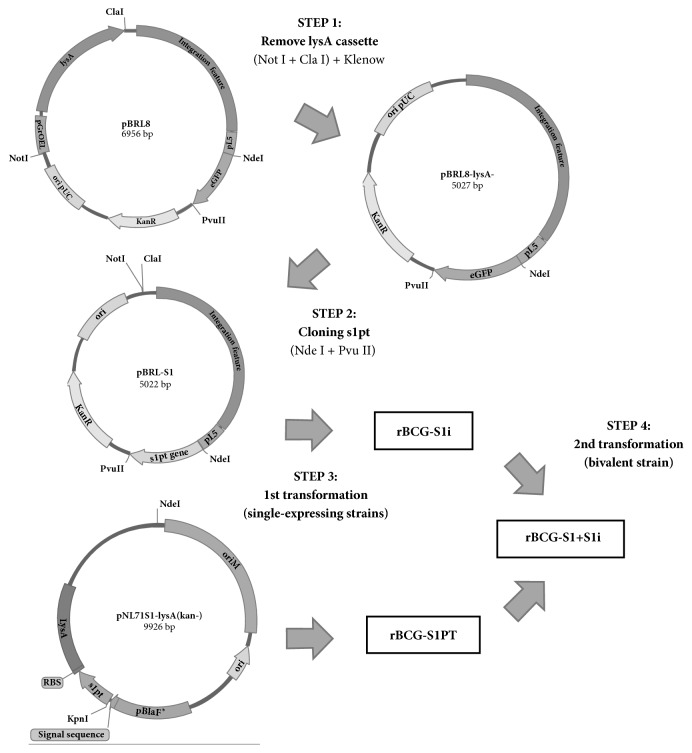
Schematic of cloning and generation of bivalent recombinant BCG strain. pBRL8 vector was digested with NotI/ClaI to remove* lysA* cassette (STEP 1) and the* s1pt* gene was PCR amplified and cloned under P_L5_ promoter thus generating pBRL-S1 vector (STEP 2). This vector was used to transform wild-type BCG (STEP 3) thus generating rBCG-S1i. In the STEP 4, rBCG-S1PT was made electrocompetent and used in a 2nd transformation step with pBRL-S1 to generate the bivalent strain.

All cloning steps were performed in* Escherichia coli *DH5*α* strain (Invitrogen) transformed by heat shock and transformants grown in LB in the presence of kanamycin (20 *μ*g/mL) for selection. Briefly, the* lysA* cassette of expression in the integrative plasmid pBRL8 was removed by digesting with Cla I and Not I, treated with Klenow and religated. Then, the genetically detoxified S1 gene sequence (*s1pt*) was PCR amplified using primers* s1-forward* 5'- TAGCATATGGACGATCCTCCCGCCACCGTATA – 3' and* s1-reverse* 5'- TAGATCGATGAACGAATACGCGATGCTTT and cloned under the regulation of the P_L5_ promoter at Nde I and Pvu II sites, thus generating pBRL-S1 (Figure 1). The correct insertion of* s1pt* was confirmed by Sanger sequencing using primer* PL5-f* 5'-TAGGTTTAAACAAACGGAAACAGCTATGACCAT-3'.

### 2.3. BCG Transformation

BCG Moreau strain was grown in Middlebrook 7H9 supplemented with OADC (MB7H9) under 5% CO_2_ at 37°C and electrocompetent cells prepared according to previous protocol [19]. Competent BCG was transformed with pBRL-S1 and clones selected by resistance to kanamycin in Middlebrook 7H10 plates supplemented with OADC and kanamycin (MB7H10). A single clone of rBCG-S1i was used to confirm S1 expression under the integrative plasmid. To generate the bivalent strain (rBCG-S1+S1i) a previously generated lysine auxotrophic BCG (rBCG-ΔlysA) complemented with pNL71S1-lysA was electroporated with pBRL-S1. In this report, the complemented auxotroph rBCG-ΔlysA-S1PT-lysA^+^ (kan−) strain, which expresses S1PT under *p*_*BlaF∗*_ promoter fused with the signal sequence of *β*-lactamase, is referred to as rBCG-S1PT.

### 2.4. Western Blotting

To confirm the expression of the S1PT antigen in rBCG-S1PT, rBCG-S1i, and rBCG-S1+S1i, the rBCG strains were grown in MB7H9 until mid-log phase when cells where centrifuged and washed twice with PBS. Cells were resuspended in PBS and lysed by sonication. Total protein extracts were separated by centrifugation into soluble and insoluble fractions and separated by SDS-PAGE. Protein extracts were electrotransferred to a PVDF membrane using a semidry electroblotter (Owl Separation Systems) and blocked with 5% nonfat dry milk solution for 16 h at 4°C. Mouse polyclonal anti-S1PT generated in-house (1:1,000) was used for antigen detection incubating the membrane for 2 h. The secondary antibody, goat anti-IgG HRP was incubated at 1:2,000 for 1 h (A6782, Sigma). Peroxidase reaction was detected using the ECL Prime Detection Reagent (GE) and a LAS4000 photoimaging equipment (GE).

### 2.5. Plasmid Stability

rBCG-S1i and rBCG-S1+S1i which possess resistance to kanamycin were grown in 5 mL of MB7H9 without antibiotic until late-log phase. 100 *μ*L of the culture was used for serial passaging up to 8 times in the same medium without antibiotic. After every passage an aliquot of the culture was used to seed MB7H10 plates with and without kanamycin. Plasmid stability was determined by the percentage of colonies able to grow in the medium with and without kanamycin.

### 2.6. Vaccine Preparation and Immunization

Single clones of wild-type BCG, rBCG-S1PT, rBCG-S1i, and rBCG-S1+S1i were grown in 50 mL of MB7H9 until mid-log phase and centrifuged. Mycobacteria were washed twice with 10% ice-cold glycerol, and aliquots were resuspended in 10% glycerol and maintained at -80°C until use. For immunization, cell concentration was adjusted to 10^7^ CFU/mL and 100 *μ*L used for intraperitoneal immunization of groups of mice (5 mice/group).

### 2.7. Phenotype and Cytokine Release of Spleen Cells

Thirty days after the single dose immunization, spleens of immunized mice were recovered and cell suspensions were prepared using a Wheaton tissue grinder. Cell suspensions (5 x 10^6^ cells/mL) were cultured in RPMI-1640 supplemented with 10% fetal bovine serum (Invitrogen) and polymyxin B (250 ng/mL). Cells were stimulated with previously prepared mycobacterial culture filtrate proteins CFP (5 *μ*g/well) [19], Concanavalin A (5 *μ*g/mL, Sigma), or left unstimulated for 48 h at 37°C in 5% CO_2_. The culture supernatants were collected and cytokine levels were quantified using the Cytometric Bead Array Th1/Th2/Th17 kit (BD Bioscience) or ELISA (Peprotech), according to manufacturer's recommendations. For phenotypical characterization of spleen cells, they were cultured for 4 h at 37°C in 5% CO2 and incubated with monensin (BD Bioscience) according to the manufacturer's instructions. Cells were fixed and stained with the following antibodies: anti-CD3-APC-Cy7, anti-CD4-PE-Cy5, TNF-*α*-FITC, and IFN-*γ*-APC, and samples were acquired on a FACSCanto II flow cytometer (BD Bioscience) and analyzed using the Flow Jo software (Tree Star).

### 2.8. Statistical Analysis

Mann-Whitney two-way Student's t test was used to evaluate the significant differences between groups for cytokine release experiments. Unpaired one-way Student's t test was used to evaluate the differences between stimulated and nonstimulated cells for the phenotypic characterization of CD4^+^ T cells.

## 3. Results

### 3.1. Single and Bivalent Expression of S1PT in Recombinant BCG

**Figure 2:**
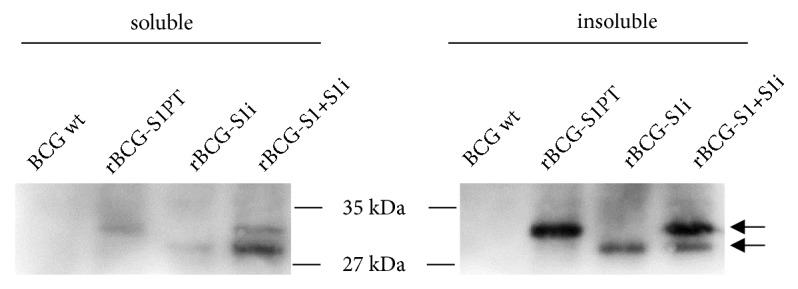
Bivalent expression of S1PT antigen in recombinant BCG. Western blot of rBCG strains expressing S1PT through distinct vectors. Protein extracts of wild-type BCG (BCG wt), rBCG-S1PT, rBCG-S1i and rBCG-S1+S1i were separated in soluble and insoluble fractions. S1PT antigen was detected using anti-S1PT previously generated in mice. The values in the center represent the molecular sizes in kilodaltons. Arrows indicate the presence of the two forms of S1PT expressed in rBCG.

The expression of S1PT in BCG through the integrative vector (rBCG-S1i) was confirmed by Western blot. Protein extracts of rBCG-S1i showed a single immunoreactive band in the expected size in both soluble and insoluble fractions (Figure 2). The rBCG-S1i strain displays a band that runs slightly lower than the rBCG-S1PT strain. The protein in the rBCG-S1PT includes a fusion of the antigen with the signal sequence of *β*-lactamase (~ 3 kDa), a feature of the expression cassette of the pNL71 vector. As expected, the expression through the integrative vector showed a lower level of S1PT than the extrachromosomal vector (Figure 2). In the bivalent construct, it is possible to distinguish two bands representing the expression of S1PT with and without the signal sequence. The protein in fusion with the *β*-lactamase's signal sequence was directed mostly to the cell wall-associated fraction, while the protein expressed from the integrative vector, without this feature, was concentrated in the intracellular fraction (Figure 2).

### 3.2. Plasmid Stability

**Figure 3:**
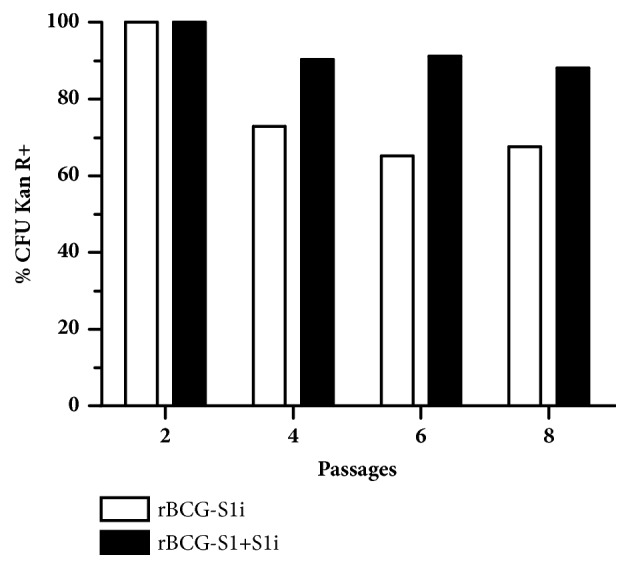
In vitro stability of bivalent rBCG construct. rBCG-S1i and rBCG-S1+S1i were serially passaged in MB7H10 plates with and without kanamycin and the percentage of CFU retaining kanamycin resistance (Kan R+) was determined according to CFU quantification.

We evaluated the stability of the integrative plasmid in the single and bivalent constructs (rBCG-S1i and rBCG-S1+S1i strains, respectively) without antibiotic pressure. Serial passages showed 67% of kanamycin-resistant colonies in rBCG-S1i throughout eight passages, while rBCG-S1+S1i displayed 88% of kanamycin-resistant colonies (Figure 3). These results indicate that the integration is relatively stable* in vitro*.

### 3.3. Cytokine Production by Splenocytes from Mice Immunized with the rBCG Constructs

**Figure 4:**
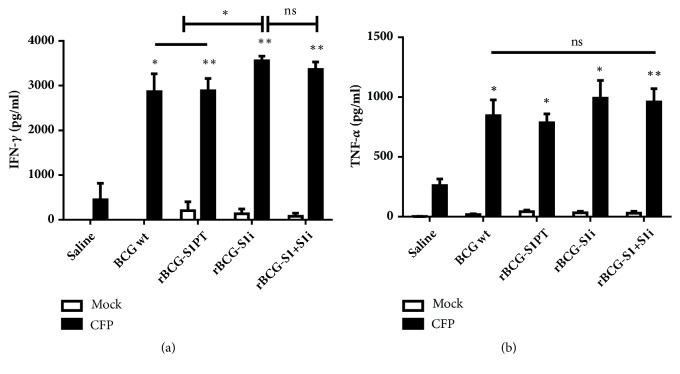
Single integrative and bivalent rBCG vaccines induce inflammatory cytokines. Splenocytes from immunized mice were cultured for 48 h in presence of CFP or medium only (mock). Cytokine levels were measured by ELISA. Data are shown as the mean (±SEM) from n = 4-5 mice per group. Statistical analyses were performed by Student's t test with a Mann-Whitney post-test. ^*∗*^*p* < 0.05, ^*∗∗*^*p* < 0.01; ns = not significant.

Groups of mice were immunized with a single dose of BCG, rBCG-S1PT, rBCG-S1i, or rBCG-S1+S1i. Four weeks later, spleen cells were isolated for quantification of TNF-*α* and IFN-*γ* production in culture supernatant following stimulation with mycobacterial proteins contained in CFP. All BCG strains induced higher levels of IFN-*γ* and TNF-*α* in comparison to the saline group (Figure 4). The rBCG-S1i group showed a significantly higher level of IFN-*γ* in comparison to wild-type BCG and rBCG-S1PT (Figure 4). IL-10, IL-4, and IL-2 were detected at very low levels (data not shown).

### 3.4. CD4^+^ T Cell Phenotype of Splenocytes from Immunized Mice

**Figure 5:**
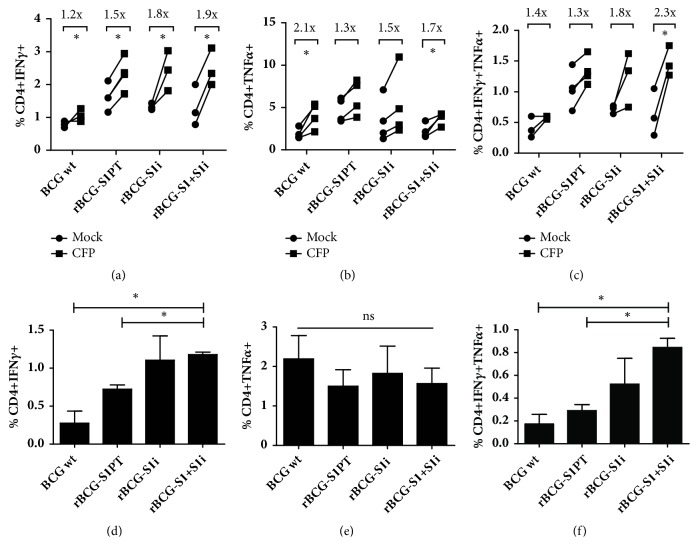
Bivalent recombinant BCG (rBCG-S1+Sli) vaccine induces higher percentage of polyfunctional CD4^+^T cells. Splenocytes from immunized mice were cultured for 18 h in the presence of CFP or medium only (mock) and stained with anti-CD3-APC-Cy7, anti-CD4-PE-Cy5, TNF-*α*-FITC and IFN-*γ*-APC for flow cytometry analysis. (A-C): percentage of CD4^+^ IFN-*γ*^+^, CD4^+^ TNF-*α*^+^ and CD4^+^ IFN-*γ*^+^ TNF-*α*^+^ T cells are expressed as a fold-change increase after stimulus. (D-F): percentage of CD4^+^ IFN-*γ*^+^, CD4^+^ TNF-*α*+ and CD4^+^IFN-*γ*^+^ TNF-*α*^+^ T cells in stimulated splenocytes from immunized mice. The bars represent the differences obtained between stimulated (in the presence of CFP) and non-stimulated (mock). ^*∗*^*p* < 0.05^*∗∗*^*p* < 0.01; ns = not significant.

To further characterize the immunogenicity induced by these vaccines, we investigated the phenotypic profile of CD4^+^ T cells recovered from the spleen through analysis of the intracellular IFN-*γ* and TNF-*α* expression. All groups of mice immunized with BCG or rBCGs strains showed a significant increase in the percentage of CD4^+^IFN-*γ*^+^ T cells when stimulated with CFP, in comparison to nonstimulated cells (Figure 5(a)). Furthermore, wild-type BCG and rBCG-S1+S1i induced a significant increase in the percentage of CD4^+^TNF-*α*^+^ T cells (Figure 5(b)). Moreover, only rBCG-S1+S1i group showed a ~ 2-fold higher percentage of double-positive CD4^+^IFN-*γ*^+^TNF-*α*^+^ T cells as compared with the nonstimulated cells (Figure 5(c)). On the other hand, the comparative analysis between CFP-stimulated groups showed that only rBCG-S1+S1i generated significantly higher CD4^+^IFN-*γ*^+^ and CD4^+^IFN-*γ*^+^TNF-*α*^+^ T cells (Figures 5(d) and 5(f)).

## 4. Discussion

There are many studies which have developed rBCGs expressing exogenous antigens from several pathogens (such as viruses, bacteria, and parasites) based on the idea of improving the strength of immune response activation through the combination of the immunological recognition against BCG (as live vector) and the foreign antigen expressed by the recombinant mycobacteria [20]. The expression of different antigens in BCG can modify the immunological properties of BCG and the antigen. Furthermore, the level of expression and localization of the antigen are also important factors in the immunogenicity induced by the strains [21]. We have obtained rBCG strains expressing the same antigen from different expression vectors, in an effort to increase the immunogenicity of the BCG strains expressing S1PT for further use as an alternative immunotherapy to murine bladder cancer model.

In this study, we showed that through the transformation of BCG with the integrative vector and the extrachromosomal plasmid, the rBCG-S1+S1i strain was indeed able to express the two forms of the S1PT antigen. Interestingly, we also demonstrated that the antigen was mainly produced by the extrachromosomal plasmid in the rBCG-S1+Sli strain. This is an expected feature since the extrachromosomal plasmid can maintain several copies per cell while the integrative vector comprises only one copy. Additionally, we also observed a distinct level of S1PT in soluble and insoluble fractions. While the extrachromosomal plasmid concentrated S1PT mainly in the insoluble fraction (cell wall-associated fraction), the integrative vector directed its expression to the soluble fraction (intracellular). This clearly indicates the functionality of the *β*-lactamase exportation signal in these constructs [22].

Another factor that can influence the immune response induced by rBCG strains is vector stability. In the absence of the antibiotic pressure, some constructs tend to lose the plasmid and consequently the expression of the heterologous antigen [21]. Our results demonstrated that the use of two expression systems did not affect the stability of the rBCG constructs. Both rBCG-S1i and rBCG-S1+S1i displayed a high proportion of kanamycin-resistant colonies, even after 8 passages in liquid culture, an indication that the integrative plasmid pBRL-S1i was present. Since the medium used to grow rBCG-S1+S1i was not supplemented with lysine, we can suppose that colonies also maintained the pNL71S1-lysA plasmid.

All groups of immunized mice showed an increased number of CD4^+^ T cells producing IFN-*γ* when stimulated with CFP. However, we observed that rBCG-S1+S1i can induce a distinct immune response. Only rBCG-S1+S1i induced an increased percentage of CD4^+^ IFN-*γ*^+^ and CD4^+^ IFN-*γ*^+^ TNF-*α*^+^ T cells, in comparison to the other rBCG strains. Polyfunctional T cells are known to be important for bladder cancer treatment. The combination of *α*-PD-1 and *α*-CTLA-4 can suppress tumor development by T cell infiltration into tumors and the induction of polyfunctional effector tumor infiltrating lymphocytes (TIL), probably CD4^+^ and CD8^+^ T cells [23]. Others demonstrated that patients with tumor recurrence showed significative reduction of CD4^+^ T cells compared to nonrecurrence patients. Moreover, the frequency of IFN-*γ* and TNF-*α*^+^ producing CD4^+^ T cells was significantly lower in patients compared to healthy controls [24], which shows that these cells are targets for bladder immunotherapy.

Our results show the possibility of immunomodulation by the bivalent expression of S1PT in rBCG, allowing enhanced Th1 immune response induced in mice, especially by the induction of polyfunctional CD4^+^ T cell responses. This study further reinforces the use of rBCG strains as an alternative in the bladder cancer therapy.

## Data Availability

The data used to support the findings of this study are available from the corresponding author upon request.
